# Relationship Between Child Turcotte Pugh (CTP) Score and Esophageal Varices (EV) Among Sudanese Cirrhotic Patients in Wad Madani Teaching Hospital, 2024: A Cross‐Sectional Study

**DOI:** 10.1002/hsr2.70918

**Published:** 2025-06-11

**Authors:** Eltype Elsiddig, Alaaeldeen Alhassan, Osman Amir, Moawia Elbalal

**Affiliations:** ^1^ Al Hasahisa Teaching Hospital Al Hasahisa Sudan; ^2^ University of Gezira Wad Madani Sudan; ^3^ Department of Hematology, Faculty of Medical Laboratory Sciences Karary University Omdurman Sudan

**Keywords:** Child Pugh score, cirrhosis, esophageal varices, Sudanese

## Abstract

**Background:**

Esophageal varices (EV) are a fatal complication of cirrhosis. The guidelines suggest that esophageal varices should be diagnosed before bleeding in all patients. In patients with cirrhosis, the Child Turcotte Pugh (CTP) class has been used as a prognostic tool for variceal bleeding.

**Aim:**

To study the correlation between CTP score and EV among cirrhotic patients.

**Methods:**

A descriptive cross‐sectional study enrolled 67 cirrhotic patients who attended Wad Madani Teaching Hospital during the period from November 2020 to November 2021. Data regarding demographics, clinical history, presentations, ultrasound findings and laboratory investigations were collected. CTP and model for end‐stage liver disease (MELD) scores were calculated for all patients. Upper gastrointestinal (UGI) endoscopy was performed and EV grades were correlated with MELD and CTP.

**Results:**

Among 67 patients, 46 (69%) were males and 21 (31%) were females, and their mean age was 52 ± 13 years. Most of the cases were CTP‐C (49%) and had MELD score from 20 to 29 (41.8%). UGI endoscopy showed Grade‐III EV in the majority of the patients (46.3%). CTP‐C and MELD scores ≥ 40 were significantly associated with advanced grades of EV (Grades III and IV) (*p*‐value < 0.0001). Also, hyperbilirubinemia, hypoalbuminemia and high INR levels were significantly associated with Grades III and IV EV (*p*‐value < 0.0001).

**Conclusion:**

The cirrhotic patients with higher MELD and CTP scores had advanced grades of EV. The study recommends that patients with Child Pugh's score B and C should have close EGD intervals due to the probability of having advance EV.

## 1 Introduction

Cirrhosis is a progressive liver condition marked by the development of fibrosis and the formation of regenerative nodules, resulting from chronic liver injury. This injury can stem from various sources, including viral infections, toxins, genetic disorders, or autoimmune diseases. Initially, the liver responds to damage by producing scar tissue (fibrosis) while maintaining its functionality. However, with prolonged injury, extensive fibrosis occurs, leading to significant loss of liver function and the eventual onset of cirrhosis. The normal lobular structure of the liver becomes disrupted as scar tissue accumulates, impairing blood flow and overall liver performance [1].

Liver disease is responsible for approximately two million deaths each year, accounting for about 4% of all global fatalities, which translates to one in every 25 deaths. Notably, around two‐thirds of these liver‐related deaths occur among men. The primary causes of mortality in liver disease are complications arising from cirrhosis and hepatocellular carcinoma (HCC), while acute hepatitis contributes a lesser share to the overall death toll. The leading factors contributing to cirrhosis worldwide include viral hepatitis, alcohol consumption, and nonalcoholic fatty liver disease [2].

Liver cirrhosis is a major health concern in Sudan, with studies indicating a significant prevalence of liver diseases attributable to various factors, including viral hepatitis and schistosomiasis. The prevalence of viral hepatitis, particularly hepatitis B and C, is alarmingly high, with estimates suggesting that about 8% of the Sudanese population is infected with hepatitis B virus (HBV) and approximately 2.2% with hepatitis C virus (HCV) [3]. Furthermore, schistosomiasis, particularly *Schistosoma mansoni* infection is as prevalent as 68.5% [4] and associated with hepatosplenic disease characterized with hepatomegaly, splenomegaly, progressive periportal fibrosis (PPF), Symmers' periportal fibrosis, and liver cirrhosis due to deposition of eggs in the small portal venules which can lead to portal hypertension and its related sequelae, mainly ascites, liver surface irregularities, esophageal varices and hematemesis [5].

In advanced chronic liver disease, hemodynamic assessment via hepatic venous pressure gradient (HVPG) reveals key thresholds: portal hypertension (PH) begins at > 5 mmHg, with mild/subclinical PH defined as 6–9 mmHg and clinically significant PH (CSPH) at ≥ 10 mmHg. Crossing the 10‐mmHg threshold markedly elevates risks for gastroesophageal varices, clinical decompensation (ascites, variceal hemorrhage, hepatic encephalopathy), postsurgical complications, and hepatocellular carcinoma. For compensated cirrhosis patients without varices, HVPG < 10 mmHg correlates with a 90% probability of avoiding decompensation over 4 years. Each 1 mmHg increase above 10 mmHg raises decompensation risk by 11%—a patient with HVPG 15 mmHg has a 55% higher risk than one at 10 mmHg when MELD and albumin levels are comparable. Severe PH (i.e., HVPG ≥ 12 mmHg) heightens variceal hemorrhage risk, while very severe PH (i.e., ≥ 16 mmHg) is linked to significantly high death rates. Despite its prognostic value, HVPG measurement remains invasive, costly, and limited in accessibility, particularly in resource‐constrained settings [6].

The integration of noninvasive methods, such as elastography and laboratory assessments, alongside the CTP score, may enhance the predictive accuracy for esophageal varices. Recent studies suggest that combining the CTP score with other clinical indicators can improve risk stratification and guide therapeutic interventions. This is particularly relevant in resource‐limited settings, where access to endoscopy may be restricted, and timely management of varices is crucial to prevent complications [7, 8].

Despite the established relationship between the CTP score and esophageal varices, there remains a paucity of data specific to Sudanese populations. Most studies have focused on Western cohorts, leaving a gap in understanding how local factors, such as schistosomiasis, influence this relationship. Therefore, this study aims to elucidate the correlation between the Child‐Turcotte‐Pugh score and esophageal varices in Sudanese patients diagnosed with liver cirrhosis. By focusing on this relationship, the research seeks to enhance understanding of the prognostic value of the CTP score in this demographic, ultimately contributing to improved clinical management and outcomes for cirrhotic patients at risk of variceal bleeding. Understanding this relationship is crucial for developing effective screening and management strategies tailored to the unique epidemiological context of Sudan, where liver disease presents a significant public health concern. This study will not only fill a gap in the existing literature but also provide a foundation for future studies aimed at improving patient care in this vulnerable population.

## 2 Materials and Methods

### 2.1 Study Design and Subjects

This descriptive cross‐sectional hospital‐based study was conducted at Wad Madani Teaching Hospital in Gezira State, Sudan, from November 2020 to November 2021. This study consecutively totally covered 67 cirrhotic patients diagnosed with portal hypertension (hepatic venous pressure gradient—HVPG ≥ 10 mmHg) [9] who presented during the specified period because of limitation number of endoscopy units in our facility and increase the strength of this study by enrolling all those patients.

The inclusion criteria included adult cirrhotic patients (≥ 18 years) based on medical history, clinical symptoms, laboratory tests, diagnostic imaging and liver biopsy in specific cases, and exclusion criteria included hemodynamically unstable patients, hepatocellular carcinoma (HCC) age more than 80 years, and patients who did not receive the required tests completely.

### 2.2 Data Collection and Patient Examination

Data were collected through structured questionnaires that included demographics, features of liver decompensation, etiologies of liver cirrhosis, ultrasound findings, laboratory investigations, and upper gastrointestinal (UGI) endoscopy. Blood samples were obtained for International Normalized Ratio (INR) and bilirubin and albumin, which were processed using a semi‐automatic biochemistry analyzer (URIT‐810).

Each patient underwent pelvi‐abdominal ultrasonography using an Aloka, Japan 5 MHz curvilinear abdominal probe to evaluate findings suggestive of cirrhosis, measure the portal vein diameter, and assess the longitudinal (bipolar) diameter of the spleen. The diagnosis of cirrhosis was based on the combination of ultrasound findings and liver function tests.

All patients underwent upper gastrointestinal endoscopy using an Olympus Evis Exera 160, Japan, performed by a Gastroenterologist of the Internal Medicine Department at Wad Madani Teaching Hospital which were blinded of patient's status. Esophageal varices categorized via Paquet's classification system that issued in 1982 [10]. Patients with microcapillaries situated in distal esophagus or oesophago‐gastric junction were classified as Grade I, one or two small varices placed in the distal esophagus as Grade II, medium‐sized varices of any quantity as Grade III, and large‐sized varices in any portion of esophagus Grade IV.

Patients with cirrhosis were classified through the Child‐Pugh score, which is depend on bilirubin, albumin, INR, ascites, and hepatic encephalopathy [1]. Serum bilirubin is estimated with a score of 1 point for levels less than 34 µmol/L, 2 points for levels fluctuating from 34 to 50 µmol/L, and 3 points for levels above 50 µmol/L. Serum albumin scored likewise, with more than 3.5 g/dL getting 1 point, levels between 2.8 and 3.5 g/dL getting 2 points, and less than 2.8 g/dL getting the extreme score of 3 points. The International Normalized Ratio (INR) is additional critical aspect, where scores are given depend on the following varieties: less than 1.7 receives 1 point, between 1.7 and 2.3 receives 2 points, and more than 2.3 receives 3 points. Additionally, the presence and severity of ascites contribute to the scoring, with absent ascites getting 1‐point, insignificant ascites grossing 2 points, and modest ascites receiving the uppermost score of 3 points. Lastly, the level of encephalopathy is evaluated, where no encephalopathy scores 1‐point, minimal encephalopathy scores 2 points, and advanced encephalopathy scores 3 points. Rendering to the Child‐Pugh Turcotte class system, all cases were characterized into Class A, B, and C when the total scores were 5 or 6, 7–9 and 10 or higher, respectively [11].

Model for end stage liver disease (MELD) score was measured based upon to the following formulation [12]:

9.57×loge(creatinine)+3.78×loge(totalbilirubin)+11.2×loge(INR)+6.43

### 2.3 Statistical Analysis

Data were entered into Microsoft Excel 2010 and analyzed using the Statistical Package for Social Sciences (SPSS; IBM, version 26.0). Results expressed as mean and standard deviation (SD) for continuous variables and number and percentages (%) for categorical variables. *χ* ^2^ test was used as a significance test for categorical variables, and ANOVA test for continuous variables. *p*‐value was considered statistically significant at a level of 0.05 (two‐sided).

### 2.4 Ethical Considerations

Ethical approval was obtained from the Internal Medicine Institutional Review Board (IRB) at the Sudan Medical Specialization Board (SMSB) (02/2021), and performed following the ethical standards of the 1964 Declaration of Helsinki [13]. Ethical approval was obtained from the authority of Wad Madani Teaching Hospital in Gezira State, Sudan. Data were used anonymously, with identity numbers instead of names, to protect patient identity. The data were kept securely in a separate file, and no reference to any individual participant was made in study reports.

## 3 Results

In total this study included 67 cirrhotic patients, 46(68.7%) were males and 21 (31.3%) were females, their mean age was 52 ± 13 years and most of them 41 (61.2%) belonged to age group from 40 to 60 years. HBV was the major etiology of cirrhosis in 28 (41.8%) cases. Ascites was reported in 50 (74.6%) and hepatic encephalopathy in 24 (35.8%) patients. Detailed baseline characteristics of the patients shown in Table 1.

**Table 1:** The baseline characteristics of the cirrhotic patients (*N* = 67).

	*N*	%
Age (Years); mean ± SD	52 ± 13	
< 40	11	16.4
40–60	41	61.2
> 60	15	22.4
Gender		
Male	46	68.7
Female	21	31.3
Etiology of cirrhosis		
HBV	28	41.8
Alcohol	7	10.4
Cryptogenic	5	7.5
AIH	4	6.0
HCV	1	1.5
NAFLD	1	1.5
Unknown	21	31.3
Ascites	50	74.6
Hepatic encephalopathy	24	35.8
Bilirubin (µmol/L)		
< 34	26	38.8
34–50	19	28.4
> 50	22	32.8
Albumin (g/dL)		
< 2.8	29	43.3
2.8–3.5	25	37.3
> 3.5	13	19.4
INR		
< 1.7	50	74.6
1.7–2.3	13	19.4
> 2.3	4	6.0

Abbreviations: AIH, autoimmune hepatitis; HBV, hepatitis B virus; HCV, hepatitis C virus; INR, international normalized ratio; NAFLD, nonalcoholic fatty liver disease.

**Figure 1:**
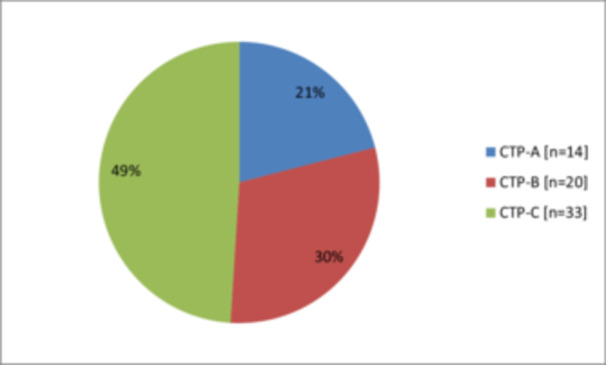
The distribution of Child‐Turcotte Pugh (CTP) classifications among cirrhotic patients (*N* = 67).

**Figure 2:**
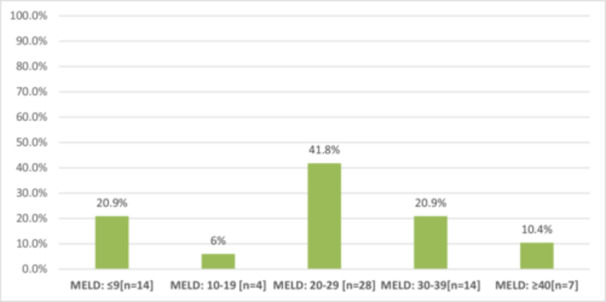
The model for end stage liver disease (MELD) score classifications among cirrhotic patients (*N* = 67).

In respect to Child‐Turcotte Pugh (CTP) classifications, 33 (49%) patients were class‐C, 20 (30%) were class‐B and 14 (21%) were class‐A as illustrated in Figure 1. Most of the cases 28 (41.8%) had MELD score from 20 to 29 (Figure 2).

**Figure 3:**
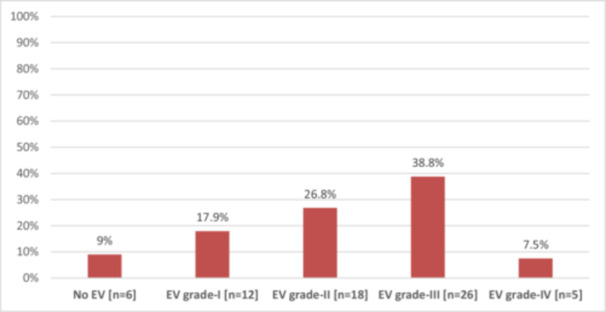
The esophageal varices (EV) grading among cirrhotic patients (*N* = 67).

Based on endoscopic examination, 26 (38.8%) patients had Grade III esophageal varices, 18 (26.8%) Grade II, 12 (17.9%) Grade I, and 5 (7.5%) had Grade‐IV esophageal varices as shown in Figure 3.

Table 2 showed that, CTP‐C classes were significantly associated with advanced Grades of EV (III and IV) (*p* < 0.0001). Likewise, Grades III and IV EV were significantly associated with higher MELD scores ≥ 40 (*p*‐value < 0.0001).

**Table 2:** The association between CTP classifications and MELD scores with EV grading.

	No EV (*N* = 6)	EV‐I (*N* = 12)	EV‐II (*N* = 18)	EV‐III (*N* = 26)	EV‐IV (*N* = 5)	*p* value
CTP						
CTP‐A	6 (100%)	6 (50%)	2 (11.1%)	0 (0%)	0 (0%)	< 0.0001^a^^,^ *
CTP‐B	0 (0%)	5 (41.7%)	12 (66.7%)	3 (11.5%)	0 (0%)	
CTP‐C	0 (0%)	1 (8.3%)	4 (22.2%)	23 (88.5%)	5 (100%)	
MELD						
≤ 9	5 (83.3%)	7 (58.3%)	2 (11.1%)	0 (0%)	0 (0%)	< 0.0001^a^^,^ *
10–19	0 (0%)	4 (33.3%)	0 (0%)	0 (0%)	0 (0%)	
20–29	1 (16.7%)	0 (0%)	12 (66.7%)	15 (57.7%)	0 (0%)	
30–39	0 (0%)	1 (8.3%)	4 (22.2%)	9 (34.6%)	0 (0%)	
≥ 40	0 (0%)	0 (0%)	0 (0%)	2 (7.7%)	5 (100%)	

Abbreviations: CTP, child‐Turcotte Pugh classifications; EV, esophageal varices; MELD, model for end stage liver disease.

^a^ *χ* ^2^ test was used.

* *p* value is significant (< 0.05).

The Spearman rank correlation showed that, the CTP scores were directly correlated with the EV grades (*r* = 0.874; *p* value < 0.0001) (Table 3).

**Table 3:** The Spearman rank correlation analysis between CTP and EV grading.

	Coefficient of variation (*r*)	*p* value
CTP vs. EV grade	0.874	< 0.0001^a^^,^ *

Abbreviations: CTP, child‐Turcotte Pugh classifications; EV, esophageal varices.

^a^ Spearman's rank correlation.

* *p* value is significant (< 0.05).

As revealed in Table 4, hyperbilirubinemia, hypoalbuminemia and high INR levels were significantly associated with advanced Grades‐III and IV EV (*p* value < 0.0001).

**Table 4:** The association between bilirubin, albumin and INR with EV grading.

	No EV (*N* = 6)	EV‐I (*N* = 12)	EV‐II (*N* = 18)	EV‐III (*N* = 26)	EV‐IV (*N* = 5)	*p* value
Bilirubin (µmol/L)						
< 34	6 (100%)	8 (66.7%)	9 (50%)	3 (11.5%)	0 (0%)	< 0.0001^a^^,^ *
34–50	0 (0%)	3 (25%)	8 (44.4%)	8 (30.8%)	0 (0%)	
> 50	0 (0%)	1 (8.3%)	1 (5.6%)	15 (57.7%)	5 (100%)	
Albumin (g/dL)						
< 2.8	0 (0%)	1 (8.3%)	1 (5.6%)	22 (84.6%)	5 (100)	< 0.0001^a^^,^ *
2.8–3.5	6 (100%)	9 (75%)	6 (33.3%)	4 (15.4%)	0 (0%)	
> 3.5	0 (0%)	2 (16.7%)	11 (61.1%)	0 (0%)	0 (0%)	
INR						
< 1.7	6 (100%)	11 (91.7%)	17 (94.4%)	15 (57.7%)	1 (20%)	< 0.0001^a^^,^ *
1.7–2.3	0 (0%)	0 (0%)	1 (5.6%)	11 (42.3%)	1 (20%)	
> 2.3	0 (0%)	1 (8.3%)	0 (0%)	0 (0%)	3 (60%)	

Abbreviations: EV, esophageal varices; INR, international normalized ratio.

^a^ *χ* ^2^ test was used.

* *p* value is significant (< 0.05).

## 4 Discussion

This study provides valuable insights into the clinical characteristics, laboratory findings, and complications of cirrhotic patients, with a particular focus on the relationship between Child‐Turcotte‐Pugh (CTP) classifications and esophageal varices (EV) grading.

The study included 67 cirrhotic patients with a male predominance (68.7%). This agreed previous study conducted in Sudan by Al Kaabi H. et al. who reported a similar male‐to‐female ratio among cirrhotic patients and attributing the higher prevalence of males to factors such as alcohol consumption and occupational hazards [14]. Internationally, the global epidemiology of cirrhosis noted a male predominance, reinforcing the notion that gender disparities exist in liver disease prevalence [15].

The mean age of our patients was 52 ± 13 years with 61.2% falling within the 40–60 age group. This finding is consistent with global trends, where liver cirrhosis commonly affects middle‐aged individuals. A study by Duah A et al in Ghana reported a similar age distribution with median age of 47 years, emphasizing that the disease often manifests in this demographic [16].

The prevalence of Hepatitis B virus (HBV) as the leading cause of liver cirrhosis in approximately half of our patients (*n* = 74; 49.3%) emphasizes the significant impact of HBV on liver health. This finding was in the range reported by Mudawi H et al among Sudanese population since a high seroprevalence of HBsAg was detected in patients with liver cirrhosis ranging from 31%–61% [3]. Also, our result was comparable to recent global trend since the HBV infection in patients with cirrhosis was 42% (35%–59%) as reported in Alberts et al. meta‐analysis [17]. In other side, Rybicka et al. reported the prevalence of HBV among cirrhotic polish patients was 21.2% [18].

In this study, 74.6% of our cases had ascites and 35.8% had hepatic encephalopathy. These findings were similar to the study of Ahmad et al. who found ascites among 75% and hepatic encephalopathy among 30.4% [19]. In the study of Seyed et al., 49.2% of the patients had ascites and 33.3% had hepatic encephalopathy [11].

Our study showed that, 49% of patients were classified as CTP‐C, 30% as CTP‐B, and 21% as CTP‐A. This distribution reflects the severity of liver disease in our context. In the study of Ahmad et al., most of the cirrhotic patients (74.6%) were CTP‐B [19].

This study found a strong association between CTP and MELD scores with the grading of EV among Sudanese cirrhotic patients. CTP‐C classes and MELD scores ≥ 40 were significantly associated with advanced EV grades (*p* value < 0.0001). Moreover, other noninvasive parameters like bilirubin, albumin and INR levels were significantly associated with high EV grades (*p* value < 0.0001). Our observations were consistent with previous studies in literature. For instance, Shrestha et al., Cherian et al. and Thapa et al. concluded that CTP scoring is a significant individual predictor of esophageal varices [20, 21, 22]. In contrary, the study of Singal and Kamath failed to show a relationship between esophageal varices with MELD and Child Pugh score [12].

This study has several limitations. First, it is a single‐center study with a relatively small sample size, which may limit the generalizability of the findings. Second, the retrospective nature of the study design may introduce potential biases and incomplete data collection. Third, we did not have an independent reviewer evaluate all endoscopic images to confirm the presence of small esophageal varices

Despite these limitations, this study has several strengths. To the best of our knowledge, this is the first study investigate the association between CTP classification and MELD scores with EV grade in cirrhotic patients in Sudan. The study provides valuable insights into the clinical characteristics and severity of cirrhosis in the Sudanese population. Additionally, the use of standardized diagnostic criteria and well‐established scoring systems, such as CTP, MELD and EV grading ensures the reliability and comparability of the findings.

## 5 Conclusion

This study concluded that, cirrhotic patients with higher MELD and Child Pugh's score had higher grades of esophageal varices leading presentation with hematemesis. Based on the findings of this study, using of MELD and Child Pugh's score as noninvasive indicator and stratification tool for EV before esophagogastroduodenoscopy (EGD) is recommended.

## Author Contributions

**Eltype Elsiddig:** conceptualization, methodology, data curation, investigation, validation, funding acquisition, visualization, project administration, resources, writing – original draft, writing – review and editing. **Alaaeldeen Alhassan:** conceptualization, methodology, investigation, validation, visualization, writing – original draft, writing – review and editing. **Osman Amir:** conceptualization, methodology, software, data curation, validation, formal analysis, writing – original draft, writing – review and editing. **Moawia Elbalal:** conceptualization, methodology, supervision, funding acquisition, investigation, validation, visualization, writing – original draft, writing – review and editing.

## Ethics Statement

Ethical approval for this study was granted by the center's ethics committee. Additionally, both verbal and written consent to publish the information were obtained from the patients.

## Consent

The authors certify that they have obtained all appropriate patient consent forms. Informed consent obtained for participation. All authors gave their approval for publication.

## Conflicts of Interest

The authors declare no conflicts of interest.

## Transparency Statement

The lead author Osman Amir affirms that this manuscript is an honest, accurate, and transparent account of the study being reported; that no important aspects of the study have been omitted; and that any discrepancies from the study as planned (and, if relevant, registered) have been explained.

## Data Availability

The data that support the findings of this study are available from the corresponding author upon reasonable request.
